# What’s New to You? Preschoolers’ Partner-Specific Online Processing of Disfluency

**DOI:** 10.3389/fpsyg.2020.612601

**Published:** 2021-01-08

**Authors:** Si On Yoon, Kyong-sun Jin, Sarah Brown-Schmidt, Cynthia L. Fisher

**Affiliations:** ^1^Department of Communication Sciences and Disorders, The University of Iowa, Iowa City, IA, United States; ^2^Department of Psychology, Sungshin Women’s University, Seoul, South Korea; ^3^Department of Psychology and Human Development, Vanderbilt University, Nashville, TN, United States; ^4^Department of Psychology, University of Illinois, Champaign, IL, United States

**Keywords:** disfluency, partner-specificity, common ground, pragmatic inference, eye-tracking

## Abstract

Speech disfluencies (e.g., “Point to thee um turtle”) can signal that a speaker is about to refer to something difficult to name. In two experiments, we found evidence that 4-year-olds, like adults, flexibly interpret a particular partner’s disfluency based on their estimate of that partner’s knowledge, derived from the preceding conversation. In entrainment trials, children established partner-specific shared knowledge of names for tangram pictures with one or two adult interlocutors. In each test trial, an adult named one of two visible tangrams either fluently or disfluently while children’s eye-movements were monitored. We manipulated speaker knowledge in the test trials. In Experiment 1, the test-trial speaker was the same speaker from entrainment or a naïve experimenter; in Experiment 2, the test-trial speaker had been one of the child’s partners in entrainment and had seen half of the tangrams (either animal or vehicle tangrams). When hearing disfluent expressions, children looked more at a tangram that was unfamiliar from the speaker’s perspective; this systematic disfluency effect disappeared in Experiment 1 when the speaker was entirely naïve, and depended on each speaker’s entrainment experience in Experiment 2. These findings show that 4-year-olds can keep track of two different partners’ knowledge states, and use this information to determine what should be difficult for a particular partner to name, doing so efficiently enough to guide online interpretation of disfluent speech.

## Introduction

Adults understand language incrementally, integrating multiple aspects of the linguistic and non-linguistic context to assign interpretations to sentences as they unfold, and to make implicit predictions about upcoming words (Tanenhaus et al., 1995; Federmeier and Kutas, 1999; Van Berkum et al., 2005). Young children do the same, as they learn the systematic patterns that structure their native language at multiple levels of analysis (e.g., Trueswell and Gleitman, 2004). For example, both adults and toddlers are quicker to look toward a named referent if its name follows a semantically constraining verb (e.g., *eat the cake* as opposed to *move the cake*; Altmann and Kamide, 1999; Fernald et al., 2008; Mani and Huettig, 2012; Gambi et al., 2016). Similarly, both adults and toddlers use the syntactic constraints of function words to anticipate what objects are about to be named (e.g., the Spanish article *la* predicts a grammatically feminine noun, and *Where are the* predicts a plural; Lew-Williams and Fernald, 2007; Lukyanenko and Fisher, 2016). Adults and children also recruit extra-linguistic information about the speaker’s goals and visual perspective into online comprehension (e.g., Clark and Wilkes-Gibbs, 1986; Schober, 1993; Nadig and Sedivy, 2002; Nilsen and Graham, 2009; Yoon et al., 2012; Heller et al., 2016; Thacker et al., 2018b).

Even disfluencies in speech guide online comprehension. Disfluencies tend to occur in predictable locations in utterances, because they often reflect speaker difficulty in sentence planning or word retrieval (Ferreira, 1991; Smith and Clark, 1993; Clark and Wasow, 1998; Clark and Fox Tree, 2002; Fraundorf and Watson, 2013). As a result, listeners can use disfluencies to predict features of the upcoming speech, such as anticipating reference to something that is difficult to describe or new to the discourse (Clark and Fox Tree, 2002; Arnold et al., 2004; Arnold et al., 2007). Even 2-year-olds show this type of disfluency effect, shifting their attention to a previously unmentioned novel object when hearing *This is thee*… *uh*… (Kidd et al., 2011). In this paper, we explore how children use disfluency to guide online comprehension, asking whether 4-year-olds’ interpretation of disfluency is influenced by their assessment of a particular speaker’s knowledge state, gathered through prior conversation.

### Disfluency

Disfluency is fairly common in casual speech, as speakers lengthen words, restart phrases, repeat words, or produce filled pauses (*um/uh*). Disfluency rates vary across contexts of speaking, and across speakers (e.g., Bortfeld et al., 2001; Clark and Fox Tree, 2002), and disfluencies of all kinds are less common in adult speech directed to very young children (e.g., 1 disfluency per 1000 words in speech to 2-year-olds, vs. 6 per 100 words in speech to adults; Fox Tree, 1995; Kidd et al., 2011). Disfluencies in speech to children become more frequent as children get older, presumably because adults address longer and more complex sentences to them (Kidd et al., 2011).

Two mechanisms have been proposed regarding how listeners come to interpret disfluencies; this work has focused on filled-pause disfluencies, with overt markers of disruption (*um/uh*). One proposal is that listeners interpret these disfluencies as signs of speaker difficulty, and base their expectations about what will come next on speaker- and context-specific inferences about possible causes of the difficulty [e.g., Clark and Fox Tree, 2002; Arnold et al., 2007, cf. inferences based on listeners’ own difficulty (Heller et al., 2015)]. Another possibility, not contradictory to the first, is that listeners might detect the predictive value of disfluencies through distributional learning (e.g., Arnold et al., 2007; Barr and Seyfeddinipur, 2010; Kidd et al., 2011). Fillers (e.g., *um/uh.*) tend to precede reference to the discourse-new. Detecting this contingency in language experience could allow listeners to anticipate appropriate referents without the need for speaker-specific inferential processing.

Previous work yields clear evidence for inferential processing in adults’ interpretation of filled-pause disfluencies (e.g., Arnold et al., 2007; Barr and Seyfeddinipur, 2010; Heller et al., 2015). For example, adult listeners expect disfluency to predict reference to a novel item that should be hard to name, but suspend this expectation if they are first informed that the speaker has a brain disorder that makes it difficult to name everyday objects (e.g., anomia; Arnold et al., 2007). Furthermore, adult listeners use their estimate of each speaker’s knowledge, as established in the previous conversation, to interpret a disfluency. For example, in a task that required communication about abstract “tangram” images (Barr and Seyfeddinipur, 2010), adult listeners anticipated reference to a new rather than a previously described object when a familiar speaker became disfluent (e.g., “um… three blobs…”), but suspended this prediction when interacting with a new partner who was unfamiliar with the tangram images. Similar effects are observed in adult multiparty conversation (Yoon and Brown-Schmidt, 2014): in dialogue, listeners look toward novel objects when a familiar speaker is disfluent (e.g., “um… it looks like… the uh… bear”). In contrast, when a third person who is unfamiliar with the objects joins the conversation, listeners’ typical expectation that disfluency predicts reference to new objects is attenuated, because the listeners attribute the speaker’s disfluency to her effort to modify the referential expression to accommodate the third person’s lack of shared knowledge. These findings show great flexibility in adults’ interpretation of filled-pause disfluencies, suggesting a role for sophisticated inferential processing about the possible reasons for disfluency in online language interpretation.

### Children’s Interpretation of Disfluency

Previous evidence suggests that children as young as 2 years of age expect disfluent descriptions (e.g., “Look at thee… um…”) to refer to a novel and discourse-new object as opposed to one that was familiar and discourse-old (Kidd et al., 2011; Morin-Lessard and Byers-Heinlein, 2019; Yoon and Fisher, 2020). This disfluency effect held when the two objects in view differed only in discourse status (both were familiar; Owens and Graham, 2016), but not when they differed only in novelty (neither had been previously named; Owens et al., 2018). Moreover, children’s predictive use of disfluencies is not limited to a simple association between disfluency and particular types of referents (e.g., discourse-new referents). Rather, like adults, children make inferences about the possible causes of disfluencies, interpreting them flexibly based on the difficulty a particular speaker should have in naming an object (Orena and White, 2015; Thacker et al., 2018a,b). To illustrate, Orena and White (2015) introduced 3.5-year-old children to either a knowledgeable speaker who competently named everyday objects, or a forgetful speaker who often could not name ordinary objects. Children who heard the knowledgeable speaker showed the expected disfluency effect, looking preferentially toward objects that were novel and discourse-new when the speaker was disfluent (e.g., “Look! Look at thee, uhh, ….”). In contrast, children who heard the forgetful speaker did not show this pattern. This result suggests that by 3.5 years of age young children can adjust their interpretation of disfluency based on known qualities of the speaker. Because they knew this speaker had trouble naming common objects, they did not treat her disfluencies as predictors of reference to something new.

In the present work, we built on these findings to probe the flexibility of children’s interpretations of disfluency. We asked for the first time whether children can track the knowledge they share with particular speakers over time, and use that assessment to interpret those speakers’ disfluencies.

### Establishment and Use of Common Ground

In order to make inferences about what might be difficult for a particular speaker to name, children must be able to track what the speaker does and does not know about the objects under discussion. Establishing and remembering what is in “common ground” with other people plays a fundamental part in communication, and considerable evidence suggests that young children keep track of the knowledge states of their interaction partners.

**FIGURE 1:**
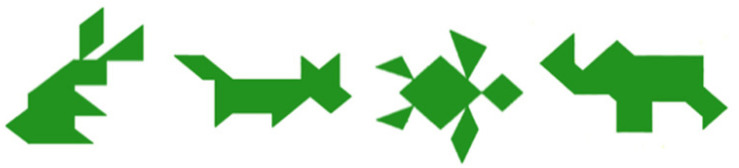
Stimuli in Experiment 1: established labels were *bunny, dog, turtle*, and *elephant* (from left to right).

For example, in conversation, interlocutors establish common ground by developing shared labels for repeatedly mentioned entities, reflecting *conceptual pacts* for how to refer to them (Krauss and Weinheimer, 1964, 1966; Clark and Wilkes-Gibbs, 1986; Isaacs and Clark, 1987; Schober and Clark, 1989; Wilkes-Gibbs and Clark, 1992; Van der Wege, 2009; Yoon and Brown-Schmidt, 2014, 2018, 2019). To illustrate, when adult speakers describe tangram images like those in Figure 1, they initially produce long, elaborated descriptions (e.g., “Find the one that looks like a dog. It has a big head and you can see two legs and a tail, two ears.”), but quickly shorten them through repeated use to develop a concise label for each image (e.g., “the dog”; Krauss and Weinheimer, 1964, 1966; Clark and Wilkes-Gibbs, 1986; Isaacs and Clark, 1987; Schober and Clark, 1989; Wilkes-Gibbs and Clark, 1992; Brennan and Clark, 1996; Van der Wege, 2009).

Once such a conceptual pact is established, interlocutors expect it to be honored (e.g., Metzing and Brennan, 2003). If a speaker introduces a new conceptualization of an object with an already-established description in common ground (e.g., shifting from “the shiny cylinder” to “the silver pipe” to describe the same novel object), listeners will be slower to understand this new description. Crucially, these conceptual pacts are partner-specific, reflecting their dependence on common ground between speaker and hearer. Listeners have less trouble understanding a new expression for an old object (“the silver pipe”) if it is introduced by *a new speaker*, one who does not share the same conversational history (Metzing and Brennan, 2003; Brown-Schmidt, 2009; see Kronmüller and Barr, 2007, 2015 for an alternative partner-independent interpretation of the timing of these effects).

Recent reports provide evidence that children, like adults, develop conceptual pacts with their interaction partners, and show signs of expecting these pacts to be partner-specific (Matthews et al., 2010; Graham et al., 2014; Köymen et al., 2014; Branigan et al., 2016). For example, during training trials, 4-year-old children heard an experimenter use a modified noun phrase (e.g., “Look at the striped ball.”) to identify a target object with two salient visual properties that could be used to identify it (e.g., a striped yellow ball; Graham et al., 2014). In later test trials, either the same experimenter or a new, naïve experimenter referred to the same object using either the original expression (e.g., “the striped ball”) or a new expression (“the yellow ball”). Upon hearing the critical noun (“ball”), children were faster to identify the target object when the same speaker used the original expression compared to when the same speaker used a new expression; this advantage for the original expression disappeared if a new speaker produced the test trials. This suggests that the children expected the original speaker but not a new speaker to uphold the established conceptual pact.

Alongside these positive findings, there are suggestions in the literature that children are less reliably sensitive to partner-specific conceptual pacts than are adults. Matthews et al. (2010), for example, though reporting evidence for partner specificity in children’s expectations regarding conceptual pacts, found that some children protested the use of a new term for an old object despite a partner change (e.g., “It’s not a pony, it’s a horse!”; see also Ostashchenko et al. (2019), for a failure to conceptually replicate Matthews et al.’s experiment). The mixed evidence on this point suggests that at least under some circumstances, children may rely on the availability of entrained labels from their own perspective, rather than successfully taking the partner’s perspective in time to guide online interpretation.

A different line of evidence suggests that much younger children make speaker-specific inferences about reference, based on keeping track of common ground with their interaction partners. In one influential study (Akhtar et al., 1996), 2-year-olds played with three novel objects along with two experimenters; the objects were never named, but were referred to using neutral terms such as “this one.” After the three objects had been experienced in this way, one experimenter left the room; in her absence, the other adult and the child played with a fourth object in the same manner. When the absent experimenter returned, she exclaimed excitedly “Look, I see a blicket”; all four objects were visible. Later comprehension tests suggested that children linked the new word with the fourth object, the one that was new to the speaker when she returned to the room. Tomasello and Haberl (2003) found a related result in 12- and 18-month-old infants’ pronoun interpretation (“Wow, Cool! Can you give it to me?”; “it” in this context was linked with the object that was new to the speaker upon her return). The infants in these studies seem to take a speaker’s excited reference to a single object as evidence that the speaker is referring to something that is new from her perspective (though all objects are old from the child’s perspective; see also Saylor and Ganea, 2007). These and many other findings support the claim that the tracking of common ground with interaction partners, knowing “what’s new to you,” plays a key role in early language development (e.g., Tomasello, 2008; Goodman and Frank, 2016). For our purposes, these findings also suggest that young children possess the social cognition tools needed to estimate their partners’ knowledge, and perhaps in turn to use that estimate to flexibly interpret their disfluent speech.

### The Present Research

In the present research, we probed the role of partner-specific inferential processing in the interpretation of disfluency. In two experiments, we manipulated speaker knowledge and tested 4-year-olds’ online processing of disfluency. In Experiment 1, the speaker was either a knowledgeable partner or a naïve partner. In Experiment 2, children interacted sequentially with two different speakers; each shared particular knowledge with the child (e.g., of either animal or vehicle tangrams). In both cases, an inferential processing account of the comprehension of disfluency would predict sensitivity to each speaker’s knowledge.

## Experiment 1

### Method

#### Participants

Forty-eight 4-year-olds (47.8–59.4 months; *M* = 51.6; 25 girls) participated in the experiment. Another ten children were excluded because of a reported language delay (1), Autism Spectrum Disorder (1), experimental errors or technical issues (6), or because they failed to complete the task (2). All were acquiring English as their native language. Children were given a book in thanks for their participation. Each child’s parent gave written informed consent, and the protocol was approved by the Institutional Review Board of the University of Illinois at Urbana-Champaign.

#### Apparatus

**FIGURE 2:**
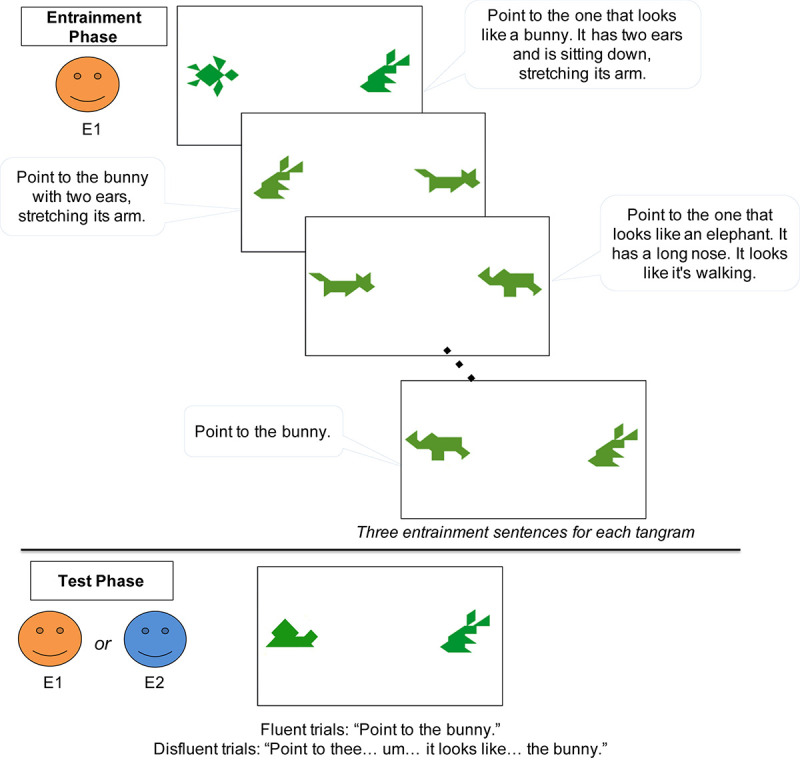
Schematic of the procedure in Experiment 1.

Children sat at a table in a brightly lit room, about 45 cm away from a 20-inch widescreen monitor. An experimenter stood behind the child and to the left, approximately 90-cm from the child’s chair. In the task, pairs of tangram images were presented on the computer screen (Figure 2). The tangram images were adapted from Yoon and Brown-Schmidt (2014). Each image was approximately 10 cm tall and 10 cm wide, and separated by 22 cm of space. A digital video camera mounted above the center of the screen recorded the child’s eye-movements (at a rate of 30 frames per second). Another camera behind the child and to the right recorded the experimental session, including the displayed images. Parents were instructed to wait outside the testing room.

#### Materials and Procedure

A child and an experimenter (E1) performed a modified version of a referential communication task (Krauss and Weinheimer, 1966). E1 first introduced the child to a secret-card game in which the child’s job was to identify the matching picture on the computer monitor based on the experimenter’s descriptions. For example, E1 said: “Today, we’re going to play a game together. You’ll have two pictures on your screen, and I have one picture on my secret card. I will tell you what I have on my card, and you find the matching picture on your screen.”

Before E1 started the game, she showed the child an example of a tangram and asked the child what the tangram image looked like. Because the tangrams were abstract images, selected not to strongly suggest a particular name, children gave various answers (e.g., a dinosaur, a key, a horse). After the child answered (e.g., “a dinosaur!”), the experimenter provided another label for the tangram that differed from child’s answer, and explained that tangrams did not have a specific name (e.g., “Yes, it could look like a dinosaur! But, to me it looks more like a key. See? Different people can have different names for this picture.”).

The experiment consisted of two phases – Entrainment and Test (see Figure 2). In the Entrainment phase, the child played the secret card game with E1. On each trial, the child saw 2 tangram images on the screen, while E1 stepped back behind the child. The experimenter described the tangram picture on her secret card, and asked the child to point to the matching picture. One tangram matched the experimenter’s secret card. The experimenter described her secret card following a script, but produced these scripted descriptions as naturally as possible. The script intentionally modeled the natural accumulation of common ground in conversation, adapted from the natural language production data collected in Krauss and Weinheimer (1964, 1966), Clark and Wilkes-Gibbs (1986), Isaacs and Clark (1987), Schober and Clark (1989), Wilkes-Gibbs and Clark (1992), Van der Wege (2009), Yoon and Brown-Schmidt (2014), Yoon and Brown-Schmidt (2018, 2019). Initially, the experimenter’s tangram descriptions were lengthy, and they became more succinct across trials as she repeatedly described the same images. For example, one tangram was first described as “*Point to the one that looks like a bunny. It has two ears and is sitting down, stretching its arm*,” but later as “*Point to the bunny*.” Across trials, the child and experimenter established entrained labels for four different tangrams that were referred to three times each (Figure 2). Children rarely made errors but on the few trials in which they pointed to the wrong image, the experimenter gave the child another chance (e.g., “Do you think so? Let’s try it again. Point to the bunny.”). Descriptions in the entrainment phase included no disfluencies.

After the Entrainment phase, E1 said “Oops, I forgot to do something today. Can I go outside and check my calendar really quick?” She then left the room briefly and checked a calendar in the waiting room (leaving the door to the experiment room open while she did so). In both speaker conditions, E1 returned and told the child that she had forgotten to pick up a package. In the same-speaker condition, E1 asked another person to pick up the package for her, so that E1 could continue the game [e.g., E1: “Oh, wait, (E2’s name), can you help me? Can you go upstairs and pick up a package for me? I have to finish the game with (child’s name).”]. In contrast, in the different-speaker condition, E1 left the lab, after asking a new experimenter (E2) to take her place [e.g., E1: “I’ll be back in 10 min, but I think (E2’s name) can play the game with you. (E2’s name), can you play the game with (child’s name)?”]. The new experimenter (E2) agreed, but made clear that she was ignorant of the game (E2: “Sure! I don’t know how to play this game but I’ll do my best.”). Speaker condition (Same vs. Different) was manipulated between subjects. The experimenters who played the roles of E1 and E2 were either one male and one female or two females. When both experimenters were female, they wore different vividly colored T-shirts to make them more distinctive.

In the Test phase, the task remained the same–the child pointed to the picture that matched the experimenter’s description of the picture on her secret card. On each test trial, there were again 2 tangrams on the screen (Figure 2). One had previously been referred to during the Entrainment phase, and the other was a tangram that the child had not seen before (thus both novel and discourse-new). All 4 critical target trials referred to the old, familiar tangram. The experimenter gave fluent instructions (e.g., “Point to the bunny.”) for half of the trials and disfluent instructions (e.g., “Point to thee… um…it looks like…the bunny.”) for the other half of the trials. The disfluent instructions were modeled after naturally produced expressions by adult participants in a prior study (Yoon and Brown-Schmidt, 2014). The experimenters were trained to produce the disfluency over a period of about 3 seconds, with each part of the disfluency (“Point to thee/um/it looks like…”) lasting approximately 1 second. The fluency of the instructions was manipulated within subjects. Regardless of fluency, the experimenter produced the same label established in the Entrainment phase (e.g., bunny). We also included 2 filler trials that described the novel and discourse-new tangram, to prevent children from ignoring novel tangrams. The experimenter gave instructions disfluently on filler trials. The location of the target object on the screen (left vs. right) was counterbalanced. During the test trials, children were not given feedback on their accuracy, and they rarely made errors.

#### Predictions

Based on previous findings with 4-year-old children and young adults (Metzing and Brennan, 2003; Graham et al., 2014), we expected that children would easily interpret fluent expressions that were previously entrained, regardless of the identity of the speaker. The key question concerned how children would process a disfluency with respect to the current partner’s knowledge state. If 4-year-old children are sensitive to partner-specific common ground and use this information while processing a partner’s disfluency, they should interpret *the same speaker’s* disfluency as indicating that the upcoming referent is novel or difficult (Arnold et al., 2004, 2007; Kidd et al., 2011). Thus, children should look at the novel/discourse-new tangram more than the familiar tangram in the same-speaker condition. The timing of this effect, specifically whether it emerges before the critical noun, would speak to the speed of this process. By contrast, in the different-speaker condition, the new experimenter is unaware of the entrained labels; thus, when the new experimenter is disfluent, the expectation that disfluency predicts a novel referent should be eliminated. If so, children’s gaze should not differ between the familiar and novel tangrams following the naïve experimenter’s disfluency.

Alternatively, if the child interprets the expressions from their own perspective, rather than that of the speaker, disfluent expressions should prompt them to look at the novel/discourse-new tangram more than the familiar one, regardless of the identity of the speaker.

#### Coding

As the critical instructions were produced live, we first marked the onset of each instruction (e.g., “Point to…”) and of the critical noun (e.g., “bunny”) to the nearest 33 ms video frame, using audio playback and a visual display of the audio waveform in Apple iMovie. The critical noun (e.g., bunny) was produced, on average, 614 ms after the onset of “Point” in fluent trials and 2,943 ms after the onset of “Point” in disfluent trials. There were no significant differences in the latency from the onset of “Point” to the onset of the critical noun across speaker conditions in either the fluent [Same Speaker: Mean = 621.5 ms (SD = 205.3), Different Speaker: Mean = 606.9 ms (SD = 118.5); *t* = −0.43, *p* = 0.67] or disfluent trials [Same Speaker: Mean = 3020.1 ms (SD = 524.3), Different Speaker: mean = 2866.7 ms (SD = 423.5; *t* = −1.58, *p* = 0.12)].

We coded children’s eye-movements during Test trials from 1 second before the onset of “Point” to 3 seconds after the onset of the critical noun. Children’s eye fixations (left, right, away, missing) were coded frame-by-frame (33 ms per frame) in iMovie. To ensure that coders were blind to condition, the coders viewed the video without sound, after the onset of each instruction had been marked in the video. If the child’s eyes were not visible, the frame was coded as “missing.” When visual fixations were coded as missing or away in more than two thirds of video frames within a critical time window defined for analysis (see Results for more details), that time window was excluded from analysis (8.3% of time windows). Reliability was assessed by a second coder for a randomly chosen 20% of the participants. The first and second coders agreed on the children’s direction of gaze for 95% of coded video frames. When the two coders did not agree, the first coder’s decision was retained.

### Results

Test trials were separately analyzed for fluent and disfluent descriptions, as the time before the critical noun was produced was significantly longer in the disfluent condition than the fluent condition (see Yoon and Brown-Schmidt, 2014).

#### Fluent Expressions

**FIGURE 3:**
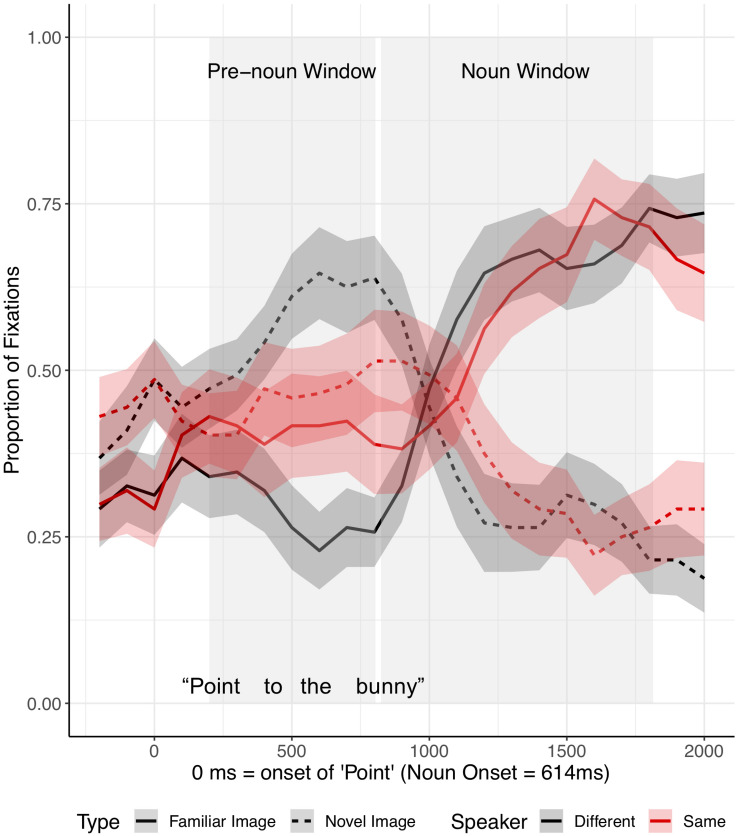
Proportion of fixations following the onset of “Point,” by Image Type (familiar vs. novel) and Speaker condition (different vs. same), for fluent trials in Experiment 1. Ribbons around the lines indicate standard errors. See Supplementary Appendix Figures 1B, 2B in Supplementary Appendix B for figures split by speaker condition.

Our primary analysis focused on children’s eye movements after the onset of “Point” (Figure 3) in two time-windows: (1) a pre-noun window extending from 200 ms after the onset of “Point” to 200 ms after critical noun onset, and (2) a noun window extending from 200 to 1200 ms after critical noun onset. The pre-noun window reflected interpretation of the description prior to the critical noun, and the noun window captured the processing of the critical noun. Both windows were offset by 200 ms, to reflect the time it takes to program and launch an eye movement (Hallet, 1986).

**FIGURE 4:**
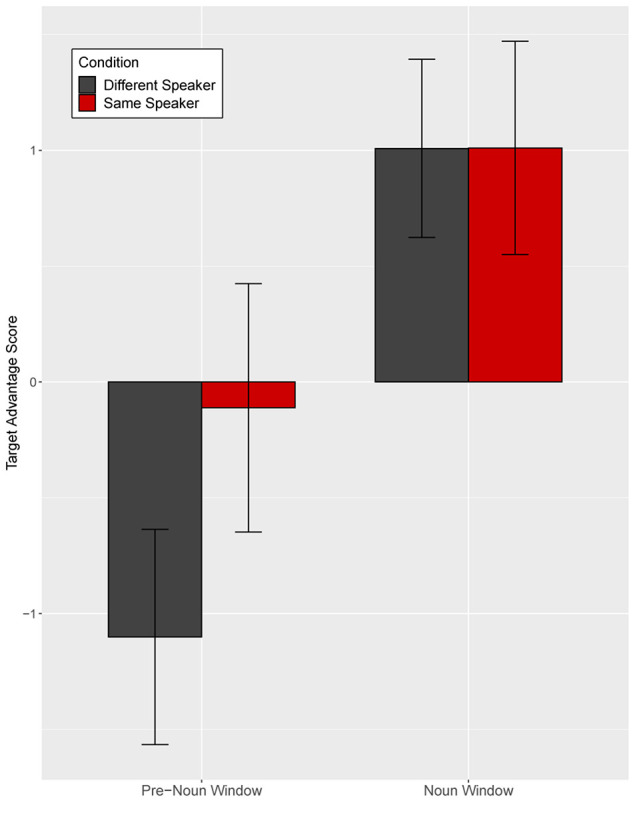
Target advantage scores for fluent trials, calculated as the log of the ratio of target to competitor fixations, in Experiment 1. Error bars indicate standard errors.

Target advantage scores were calculated as the empirical logit for the ratio of target fixations (fixations to familiar tangrams) to distractor fixations (fixations to novel tangrams) and used as the dependent measure (Figure 4). A target advantage score of zero indicates no preference between target and distractor, while positive values indicate a target preference and negative values indicate a distractor preference. In the pre-noun window, children looked at the distractor (novel image) more than the target image (familiar image), with this novelty preference appearing somewhat larger in the different-speaker than in the same-speaker condition. This could suggest that they expected the same speaker to continue to refer to familiar images. In the noun window, children correctly identified the target upon hearing the critical noun.

We analyzed the data in a mixed effects model with a Gaussian link function with subjects and items as random intercepts. The model included speaker identity (same vs. different speaker) and time window (pre-noun vs. noun window) as fixed effects, which were coded with mean-centered Helmert contrast codes (see Supplementary Material for full model details; Supplementary Appendix Table 1). The model revealed only a main effect of time window as target fixations increased over time within the trial (*t* = −4.22, *p* = 0.004). The identity of the speaker did not affect interpretation of fluent expressions (*t* = −0.003, *p* = 1.00).

Separate planned analyses of each window were performed, although the interaction between speaker identity and time window was not significant (*t* = 1.18, *p* = 0.27). In the pre-noun window, children looked numerically more at the novel tangram (the distractor) in the different-speaker than in the same-speaker condition; this effect was not significant (*t* = −1.69, *p* = 0.10, see Figure 4). Looking patterns in the noun time-window did not differ across speaker conditions (*t* = 0.02, *p* = 0.98). These results show that children interpreted fluent expressions without delay in both conditions.

#### Disfluent Expressions

**FIGURE 5:**
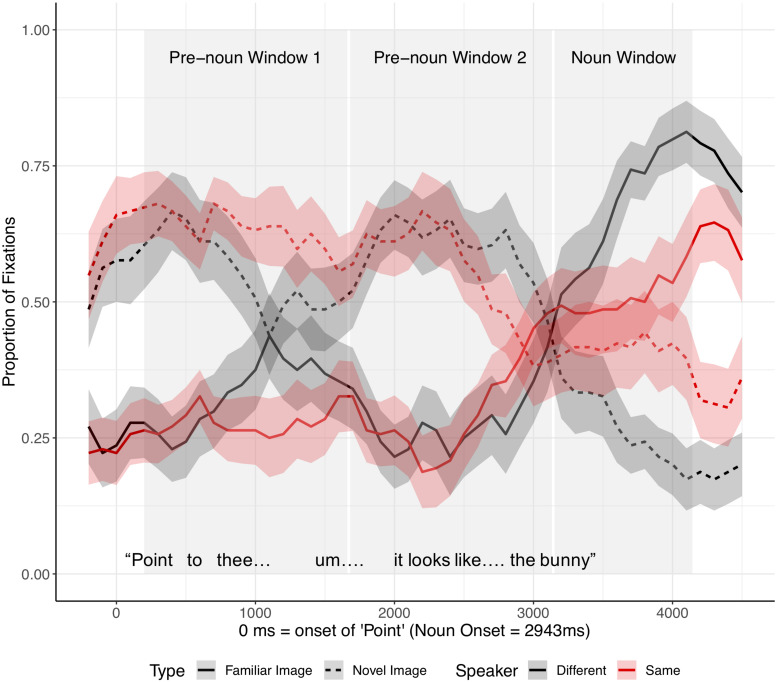
Proportion of fixations following the onset of “Point,” by Image type (familiar vs. novel image) and Speaker condition (different vs. same), for disfluent trials in Experiment 1. The target was always an old, familiar tangram that participants had seen during Entrainment, while the distractor was always a novel tangram. Ribbons around the lines indicate standard errors. See Supplementary Appendix Figures 3B, 4B in Supplementary Appendix B for figures split by speaker condition.

As in the analysis of fluent expressions, we analyzed the children’s eye movements from the onset of “Point.” Critical noun onset occurred, on average, 2,943 ms after the onset of “Point” (Figure 5). Since the time between the onset of “Point” and the onset of the critical noun was longer for disfluent instructions than fluent instructions, we analyzed the eye movements in three time-windows: (1–2) two equally divided pre-noun windows extended from 200 ms after the onset of “Point” to 200 ms after noun onset (pre-noun window 1: 200–1,671 ms after the onset of “Point” vs. pre-noun window 2: 1,671–3,143 ms after the onset of “Point”), and (3) the noun window extended from 200 to 1200 ms after noun onset. The two pre-noun windows captured how children interpreted the disfluency, and the noun window reflected the processing of the critical noun phrase.

**FIGURE 6:**
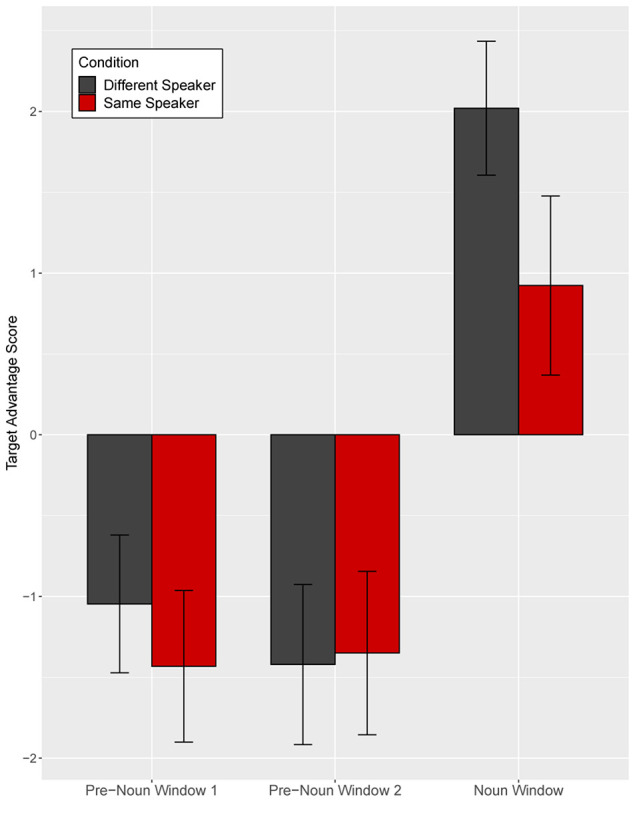
Target advantage scores for disfluent trials, calculated as the log of the ratio of target to competitor fixations, in Experiment 1. Error bars indicate standard errors.

Target advantage scores were calculated as before (Figure 6). Before hearing the critical noun, children looked more at the (novel) distractor than at the target image in both speaker conditions. In the noun window, children in the different-speaker condition showed a stronger target advantage than did children in the same-speaker condition; thus, children identified the target image faster following a disfluency when they interacted with a different speaker as opposed to with the same speaker who participated in entrainment.

Target advantage scores were analyzed as before (Supplementary Appendix Table 2). Time window was coded with two Helmert contrasts. The first contrast (Window 1) tested the difference between the two pre-noun windows and the noun window, and the second contrast (Window 2) tested the difference between the first and the second pre-noun windows. The omnibus model revealed a significant main effect of Window 1, indicating increased target fixation across time within the trial (*t* = −9.89, *p* < 0.0001). Neither the main effect of speaker identity (*t* = −1.35, *p* = 0.18) nor the effect of Window 2 (*t* = 0.42, *p* = 0.67) was significant. The interaction between speaker identity and Window 1 was marginally significant (*t* = 1.76, *p* = 0.08), but the interaction between speaker identity and Window 2 was not significant (*t* = −0.76, *p* = 0.45).

Separate planned analyses of each window were performed, although the interaction between speaker identity and Window 1 was only marginally significant. During the two pre-noun windows, there was no evidence that the identity of the speaker influenced children’s processing of a disfluency (pre-noun window 1: *t* = −0.83, *p* = 0.41; pre-noun window 2: *t* = 0.22, *p* = 0.83, see Figure 6). Children tended to look at the novel tangram more than the familiar tangram, regardless of speaker, before hearing the critical noun. A significant effect of speaker identity emerged in the noun window, such that children looked significantly less at the target (familiar) tangram in the same-speaker than in the different-speaker condition (*t* = −2.23, *p* = 0.03).

This result suggests flexibility in children’s online interpretation of disfluency. When interacting with their original partner, children expected disfluent descriptions to refer to new referents, and had to overcome this (violated) prediction when they heard the critical noun (e.g., following the disfluency, the noun “bunny” referred to the familiar image); they were slower or less likely to switch their gaze from the novel referent to the familiar referent. In contrast, children did not expect the new speaker’s disfluencies to refer to novel referents, suggesting that they attributed the disfluency to the new speaker’s lack of familiarity with both images. In conversation, children consider what is in common ground *with a particular speaker*, and use that knowledge to interpret what speakers say and how they say it.

### Discussion

The results of Experiment 1 provide preliminary evidence that children are sensitive to common ground held with the current partner, and interpret disfluency with respect to the current speaker’s knowledge. In the disfluent trials, children identified the target more readily when the new experimenter was disfluent compared to when the same experimenter was disfluent. This finding suggests that children’s referential processing was disrupted when the familiar speaker referred disfluently to a familiar referent; in contrast, the new speaker’s disfluent speech could be attributed to the speaker’s lack of knowledge.

This partner effect, however, emerged in the noun window, a delay compared to findings from previous studies with young adults, for whom the comparable effect emerged prior to the noun (Yoon and Brown-Schmidt, 2014). Similarly, prior work with 2- to 4-year old children also revealed sensitivity to disfluency prior to the noun (Kidd et al., 2011; Orena and White, 2015; Yoon and Fisher, 2020). One possible explanation for the delayed effects of partner on disfluent trials is that children are less efficient at taking another’s perspective in online language processing (see Epley et al., 2004). Alternatively, it may be that the children are about as efficient as adults, but the presence of the novel tangram in the test trials captured their attention, delaying the emergence of the partner effect in our data. However, another alternative explanation of the children’s apparent partner-sensitivity in Experiment 1 is that the “disfluency = new” association was attenuated in the different-speaker condition because the introduction of the new partner signaled an abrupt context change, potentially slowing memorial access to which items were old or new from the child’s own perspective (see Smith and Vela, 2001; Brown-Schmidt et al., 2015). One way to address these alternative interpretations is to create experimental situations in which we can test for the partner specificity of interpretation while holding constant experimenter and tangram familiarity. This is the aim of Experiment 2.

## Experiment 2

In Experiment 1, children successfully learned image labels with a partner in conversation and used this partner-specific information when they processed disfluent speech, although the key interaction of speaker and time-window was marginal. In Experiment 2, we provided a stronger test of partner-specificity by asking if children could develop distinct representations of common ground held with different partners and use these representations to guide online language processing in a partner-specific manner. In Experiment 2, in the test trials, both of the tangrams were familiar to the child, but only one of them was familiar to the speaker. We accomplished this by establishing names in entrainment trials for two sets of tangrams: animal tangrams were entrained with one experimenter, and vehicle tangrams were entrained with the other experimenter. At Test, the children viewed one animal and one vehicle tangram on each trial. Critically, while both tangrams were familiar from the child’s perspective, only one of the tangrams was familiar to the experimenter. We tested the children’s on-line processing of disfluency to examine if they interpreted the speaker’s disfluency based on the current speaker’s knowledge state, rather than on their own knowledge.

### Method

#### Participants

Forty-eight 4-year-olds (48.0–59.2 months; *M* = 52.0; 25 girls) participated in the experiment. Another eleven children were excluded because of a reported autism spectrum diagnosis (1), cerebral palsy (1), experimental errors or technical issues (3), insufficient eye movement data^1^ (3), or because they failed to complete the task (3). All were acquiring English as their native language. None of the children in Experiment 2 had participated in Experiment 1.

#### Materials and Procedure

**FIGURE 7:**
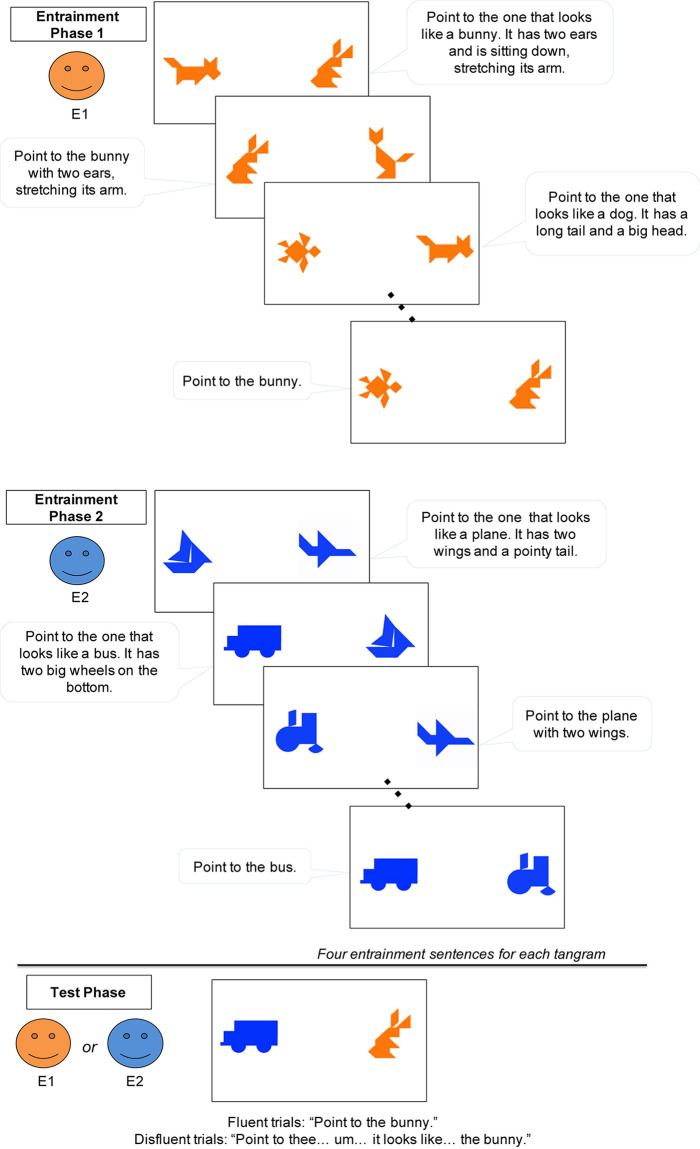
Schematic of the procedure in Experiment 2.

The materials and procedure of Experiment 2 were similar to those of Experiment 1 except for the following changes: Experiment 2 consisted of three phases rather than two – two separate Entrainment phases followed by a Test phase (see Figure 7). In each of the two entrainment phases, the child interacted with one of two experimenters (first E1 then E2). Each experimenter introduced a set of novel tangrams that belonged to either an animal category or a vehicle (things-that-go) category. Thus across the two entrainment phases, one experimenter was associated with animal tangrams and the other was associated with vehicle tangrams. The tangram images from the two categories also differed in color (animal tangrams were orange and vehicles were blue), in order to better distinguish them (see Yoon et al., 2019 for a similar technique). At Test, one of the experimenters (either E1 or E2) continued to play the card game with the child, in which the child was shown an animal tangram and a vehicle tangram that they had previously seen during entrainment. Finally, we increased the number of entrainment trials within each phase (from 12 to 16) to help children establish partner-specific common ground with the two individuals. We also increased the number of test trials (from 4 to 8) to increase statistical power in Experiment 2. As in Experiment 1, the Experimenters were either one male and one female, or two females. When the Experimenters were two females, in order to better distinguish them, they wore two different vividly colored T-shirts.

In the first entrainment phase, E1 described the picture on her secret card and the child pointed to the matching picture on the screen. The child was shown tangrams of one category, either animals or vehicles (which category came first was counterbalanced across children). As in Experiment 1, the experimenter’s descriptions shortened across trials, establishing a single-word label for each tangram by the last trial (e.g., “bunny”). There were four tangrams in each category that were repeated four times each; thus each child completed a total of 16 trials in each Entrainment phase. After the first Entrainment phase, E1 left the room [e.g., E1: “That’s it for me. It was really cool! I’ll switch with (E2’s name).”] and E2 entered the room to continue the game. The entrainment trials with E2 were identical, except that E2 now described four tangrams from the other category.

Following the two Entrainment phases, either E1 or E2 performed the Test phase (saying “It’s my turn again! Let’s finish the game.”). Before the test started, the experimenter reminded the child that she knew the tangrams from one category but not the tangrams from the other category [e.g., “Now, I have both orange animals and blue things-that-go. I had played the orange animals (or blue things-that-go) with you, so I know them very well. However, I don’t know what the blue things-that-go (or orange animals) are. I’ll try my best.”]. During the Test, the child viewed one animal and one vehicle tangram on the screen. The critical within-subjects manipulations at test were the experimenter’s fluency (fluent vs. disfluent) and the experimenter’s familiarity with the target (familiar vs. unfamiliar to the speaker). Note that from the child’s perspective, both of the tangrams were familiar, whereas from the experimenter’s perspective, only one of the tangrams was familiar. There were a total of 8 Test trials; half of the trials were fluent and half were disfluent. The order of the two Entrainment phases (animal vs. vehicle tangrams), the identity of the speaker during Test (E1 vs. E2 from Entrainment), and the location of the target object on the screen (left vs. right) were all counterbalanced across participants.

Following the test phase, the child performed a memory test. The experimenter who did not participate in the Test showed the child all 8 tangram cards at once – 4 animal and 4 vehicle cards. The child was asked to pick out the cards that the current experimenter knew.

#### Predictions

Consistent with the results of Experiment 1, we expected children to readily identify the target object when the description was fluent, regardless of target type or speaker’s knowledge state. In Experiment 2, we held constant the familiarity of the tangrams and asked if children could take into consideration the current speaker’s perspective when interpreting disfluent expressions. If they do take the speaker’s knowledge into consideration in processing her disfluency, then children should look more at the object that is unfamiliar to the current speaker when they hear a disfluent description. If so, we would expect positive target advantage scores when the target is unfamiliar to the speaker and negative target advantage scores when the target is familiar to the speaker. The timing of this effect will speak to the question of whether children’s use of perspective is delayed (Epley et al., 2004).

Alternatively, if children do not consider the speaker’s knowledge but rely on their egocentric knowledge when processing disfluency, we would expect an equivalent pattern of fixations, regardless of target familiarity.

#### Coding

**FIGURE 8:**
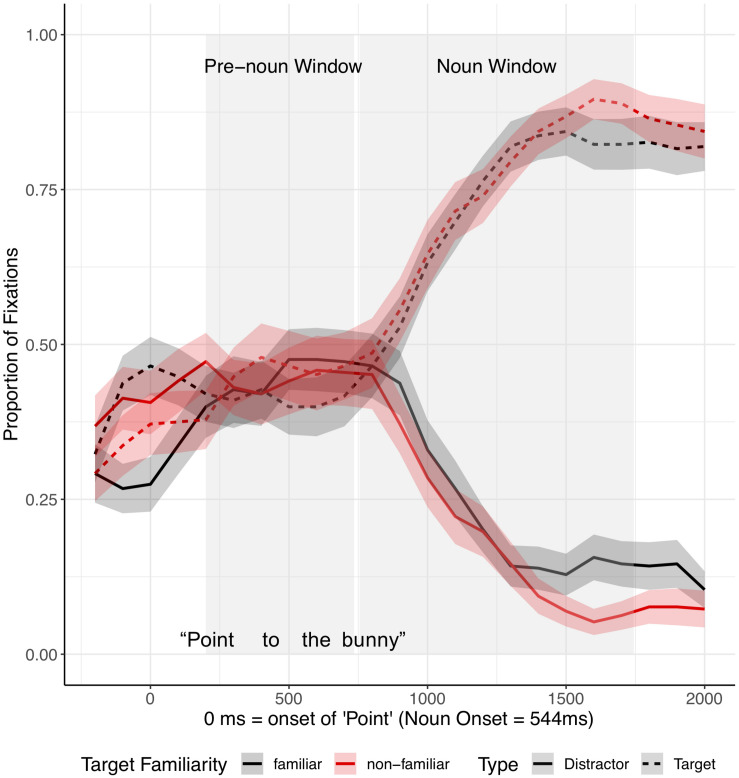
Proportion of fixations after the onset of “Point,” by Image type (distractor vs. target) and Target Familiarity (familiar vs. unfamiliar from the speaker’s perspective), for fluent trials in Experiment 2. Ribbons around the lines indicate standard errors. See Supplementary Appendix Figures 5B, 6B in Supplementary Appendix B for figures split by target familiarity.

**FIGURE 9:**
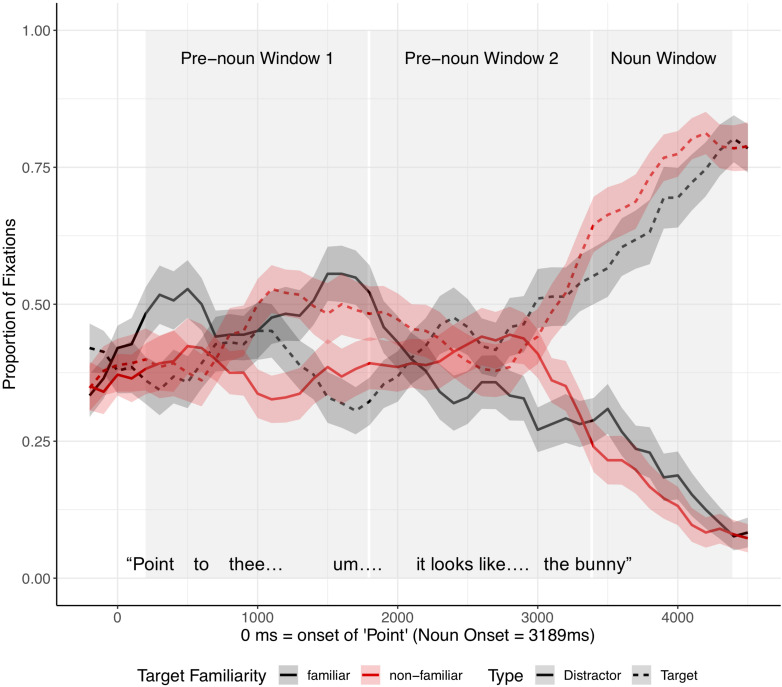
Proportion of fixations after the onset of “Point,” by Image type (distractor vs. target) and Target Familiarity (familiar vs. unfamiliar from the speaker’s perspective), for disfluent trials in Experiment 2. Ribbons around the lines indicate standard errors. See Supplementary Appendix Figures 7B, 8B in Supplementary Appendix B for figures split by target familiarity.

As in Experiment 1, we first marked the onset of “Point” and of the critical noun (e.g., bunny) in each trial, as the instructions were produced live by the experimenter. The latency of the critical noun after the onset of “Point” was on average 543.58 ms in the fluent trials and 3189.24 ms in the disfluent trials (see Figures 8, 9). There was no significant difference in the latency of the critical noun across conditions familiar (F) vs. unfamiliar (UF) object from the experimenter’s perspective] for either fluent [F: Mean = 539. 2 ms (SD = 119.4); UF: Mean = 547.9 ms (SD = 115.8); *t* = −0.51, *p* = 0.61] or disfluent expressions [F: Mean = 3170.9 ms (SD = 690.8); UF: Mean = 3241.3 ms (SD = 633.3); *t* = −0.78, *p* = 0.43].

Children’s eye-movements during the Test phase were coded manually using the same procedure as in Experiment 1. 8.0% of the time windows were excluded from analysis because more than two thirds of video frames were coded as missing or away. Reliability between the coders was high (96%, assessed for 20% of the participants). Performance on the memory test was coded as a binary measure – whether each tangram was correct or not. If the child chose four tangrams from the same category that the experimenter had previously described and none from the other category, all 8 tangrams were coded as correct and accuracy was 8/8. If the child missed one tangram from the correct category and replaced it with another tangram from the other, incorrect category, then we coded those 2 cards as incorrect, and accuracy was 6/8.

### Results

As before, we analyzed fluent and disfluent trials separately, because of the large differences in latency between disfluent and fluent trials.

#### Fluent Expressions

**FIGURE 10:**
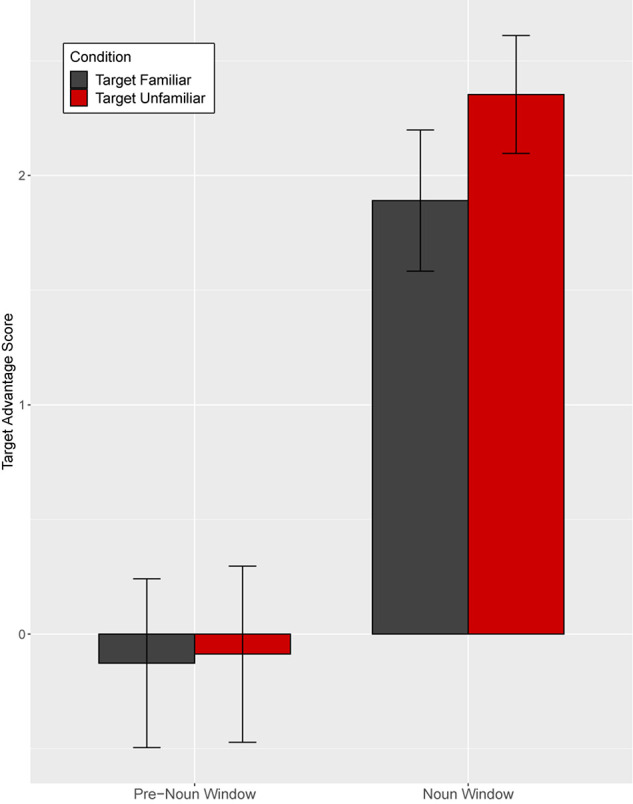
Target advantage scores for fluent trials, calculated as the log of the ratio of target to competitor fixations, in Experiment 2. Test trials were separated based on target familiarity (whether the target was familiar vs. unfamiliar from the speaker’s perspective). Error bars indicate standard errors.

Eye movements in response to fluent expressions were analyzed in two time-windows: (1) a pre-noun window, from 200 ms after onset of “Point” to 200 ms after critical noun onset, and (2) a noun window, from 200 to 1200 ms after noun onset. As in Experiment 1, the dependent measure, target advantage score, was calculated as the empirical logit of the ratio of target fixations to competitor fixations (Figure 10). As Figure 10 shows, looks to the distractor and target were approximately equal in both conditions during the pre-noun window. Upon hearing the critical noun, children looked at the target image more than the distractor image.

A mixed-effects model with a Gaussian link function included target familiarity (familiar vs. unfamiliar object from the experimenter’s perspective) and time window as fixed effects (Supplementary Appendix Table 3). The model revealed a significant main effect of time window (*t* = −7.26, *p* < 0.0001), but the main effect of target familiarity (*t* = 1.36, *p* = 0.18) and the interaction between time window and target familiarity (*t* = −0.95, *p* = 0.35) were not significant. Planned comparisons for each time window did not show significant effects of target familiarity on target advantage scores (pre-noun window: *t* = −0.02, *p* = 0.99; noun window: *t* = 1.41, *p* = 0.17).

In sum, for fluent expressions, children showed no clear preference for either object prior to the noun. Then, upon hearing the critical noun, children quickly identified the target. This finding is consistent with the results of Experiment 1.

#### Disfluent Expressions

**FIGURE 11:**
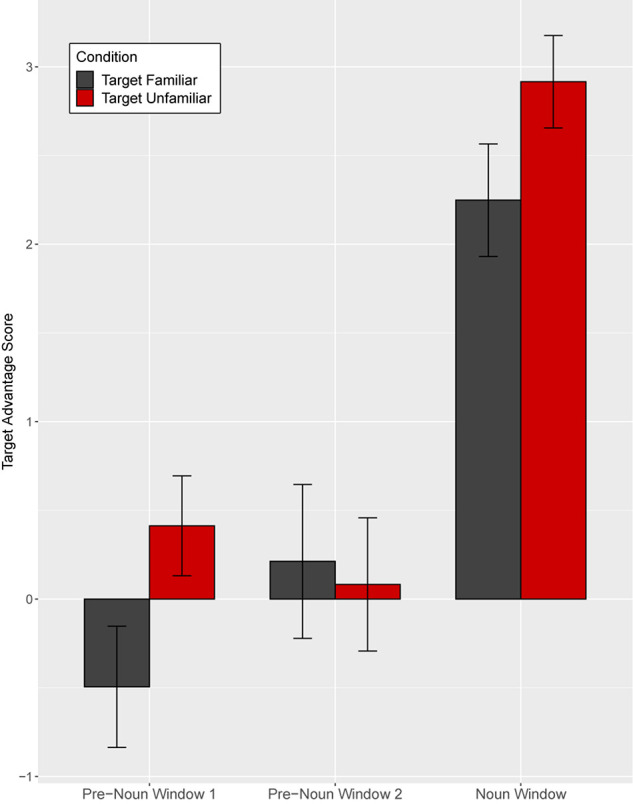
Target advantage scores for disfluent trials, calculated as the log of the ratio of target to competitor fixations, in Experiment 2. Test trials are separated based on target familiarity (familiar vs. unfamiliar object from the speaker’s perspective). Error bars indicate standard errors.

As in Experiment 1, we analyzed the eye gaze data in the disfluent trials in three time-windows (Figure 11): (1–2) two equally divided pre-noun windows from 200 ms after onset of “Point” to 200 ms after noun onset, and (3) a noun window from 200 to 1200 ms after noun onset.

As Figure 11 shows, in the first pre-noun window, target advantage scores differed across the two target familiarity conditions, indicating that in both target conditions children looked more at the image that was unfamiliar from the speaker’s perspective. That is, when the target was familiar from the speaker’s perspective, the target advantage score was negative, showing anticipatory looks to the distractor (which was unfamiliar to the speaker). When the target was unfamiliar to the speaker, the target advantage score was positive, showing anticipatory looks to the target. Note that children did not yet know which image was the target in the pre-noun windows. In the second pre-noun window, children looked about equally at the distractor and the target. Lastly, in the noun window, children were faster to identify the target when the target was unfamiliar as opposed to familiar from the speaker’s perspective.

A mixed-effect model included target familiarity (familiar vs. unfamiliar target from the speaker’s perspective) and time window as fixed effects. Time window was coded with Helmert coding. The first contrast (Window 1) tested the difference between the two pre-noun windows and the noun window and the second contrast (Window 2) tested the difference between the first and the second pre-noun window. This model revealed a significant main effect of target familiarity (*t* = 2.24, *p* = 0.03) and Window 1 (*t* = −12.23, *p* < 0.001), and a significant interaction between target familiarity and Window 2 (*t* = 2.10, *p* = 0.04) (Supplementary Appendix Table 4). The main effect of Window 2 (*z* = −0.79, *p* = 0.43) and the interaction between target familiarity and Window 1 (*z* = −0.63, *p* = 0.53) were not significant. Planned comparisons revealed a significant effect of target familiarity in the first pre-noun window (*t* = 2.70, *p* = 0.01), showing that children looked more at the object that was unfamiliar from the speaker’s perspective from early stages of disfluency processing. This effect was not significant in the second pre-noun window (*t* = −0.39, *p* = 0.70), but it re-emerged during the noun window (*t* = 2.21, *p* = 0.03). This late emerging effect upon hearing the critical noun was consistent with the results in Experiment 1.

These results suggest that children successfully attributed the speaker’s disfluency at test to that speaker’s attempt to refer to an unfamiliar tangram. This effect emerged in the first half of the disfluency, disappeared during the second half of the disfluency, and then re-emerged during the noun window. The disfluencies in this study, approximately 3200 ms, were longer than disfluencies used in previous studies (e.g., ∼2 s in Kidd et al., 2011), which may have contributed to the disappearance of the effect in the second time-window.

#### Memory Test

Accuracy on the memory test was good overall, 6.27/8 on average (range: 0/8–8/8; Median = 8; SD = 2.63), indicating that the children successfully recalled which partner was associated with which set of tangrams.

### Discussion

We found that 4-year-old children were able to learn two distinct representations of common ground with different partners, and use these representations when later encountering a partner’s disfluent speech. Children attributed the disfluency to the current speaker’s lack of knowledge, and therefore directed anticipatory looks toward the tangram that was unfamiliar to the speaker during the disfluency period. Because all tangrams were familiar from the children’s own perspective, this result is strong evidence that children successfully took their partner’s knowledge state into consideration, rather their own knowledge, in online interpretation of a disfluency. This partner-specific interpretation of disfluency as signaling reference to new information appeared early in the processing of the speaker’s expressions. The fact that Experiment 2 employed a design in which both tangram categories and both Experimenters were familiar to the children may have supported their early use of this information. Children’s memory performance was generally good, showing that children typically remembered what information had been shared with whom in our context.

## General Discussion

The results of two experiments show that 4-year-old children can successfully represent two different partners’ knowledge states and use this information appropriately when interpreting disfluency in live conversation. This finding demonstrates that 4-year-old children process disfluency based on their estimate of their partner’s knowledge, rather than on their egocentric knowledge.

In Experiment 1, when interpreting a disfluent expression, children tended to look at the novel and discourse-new object rather than the familiar object when they interacted with the knowledgeable speaker, but not when they interacted with the naïve speaker. This pattern must be interpreted with caution because the key interaction was marginally significant, but these signs that children’s interpretation was tailored to the knowledge of the speaker show sensitivity to speaker perspective. This finding points to a role for inferential processing in the interpretation of disfluency, as opposed to simple associations between disfluency and novel/discourse-new referents. In Experiment 2, children viewed two familiar objects, only one of which was unfamiliar and discourse-new to the speaker. When interpreting disfluent expressions, children showed an early preference to gaze at the image that was unfamiliar to the speaker, much as adults do (Arnold et al., 2007; Yoon and Brown-Schmidt, 2014). Taken together, the current findings provide further evidence that children’s interpretation of disfluent speech is speaker-specific, suggesting that children make rapid and sophisticated inferences about a speaker’s use of disfluency based on the current speaker’s knowledge state in live conversation.

The results of Experiment 2 are particularly striking. The current findings show that children can develop two distinct representations of common ground while communicating, and flexibly retrieve them to guide online processing of disfluent speech. This is consistent with recent findings demonstrating 4-year-old children’s sensitivity to two speakers’ differing visual perspectives during online reference resolution (Khu et al., 2020). Just as 4-year-olds consider what objects each speaker can or cannot see when interpreting her words, our findings show they also consult their memory to determine what objects are known or unknown to each speaker from prior conversation.

A possible alternative interpretation of the current findings is that the phrase “… looks like…” in disfluent test trials, independent of other features of the extended disfluency, might have reminded children of the first round of entrainment, suggesting that the speaker was naming a new referent. The extended disfluencies in our experimental scripts were intentionally modeled on naturally produced instructions in an earlier study (Yoon and Brown-Schmidt, 2014). These utterances therefore have high ecological validity, but the fact that they included multiple types of disfluency (e.g., elongated “the,” filled pauses, and “looks like”) makes it difficult for us to tell which type of disfluency drove the effects in our current studies. However, we can offer two considerations that help rule out this alternative interpretation. First, although the disfluent test instructions and the first instruction in the entrainment phase both included “looks like,” they differed in other respects. In particular, the entrainment trials included no filled pauses or elongated definite articles (e.g., thee … um). These markers of disfluency were first introduced during the test trials. Second, as shown in Figures 10, 11, in Experiment 2 children showed their disfluency effect (looking at the image that was unfamiliar to the current speaker) in pre-noun window 1 of the extended disfluency, before they heard the phrase “looks like.” This suggests that other markers of disfluency (“thee … um …”) drove our key effects.

Several factors may have contributed to children’s success in tracking and using partner-specific common ground online in our task. First of all, we used abstract tangram images that did not invite obvious names, rather than easily namable everyday objects. Tangrams are often used in studies of common ground in conversation for exactly this reason, because they lack conventional labels that any competent speaker would be expected to know (Krauss and Weinheimer, 1964, 1966; Clark and Wilkes-Gibbs, 1986; Wilkes-Gibbs and Clark, 1992; Yoon and Brown-Schmidt, 2014). As noted earlier, in a prior study of children’s understanding of conceptual pacts that used everyday objects (e.g., horse/pony) as stimuli, some children complained when even a new speaker used a new label (Matthews et al., 2010). Children may have interpreted the experimenter’s initial labeling of each object as information about its conventional category label. In contrast, children in our studies collaborated with the experimenters to label less codable tangram images across a series of entrainment trials. This process, and the relative difficulty of describing these abstract images, may have alerted children to the need to consult their partners’ knowledge states.

Another factor that might have supported children’s success in our tasks is that we provided additional cues in both experiments that might have alerted children to the need to consult partner-specific common ground. These included the new experimenter’s verbal statement before the test trials in Experiment 1 that she was not familiar with the tangram images, and the category and color differences between the tangrams shared with each interlocutor in Experiment 2 (e.g., orange animals vs. blue vehicles). Access to the appropriate memory representations is required to use jointly shared knowledge in conversation; failures of memory may cause poor perspective-taking (Horton and Gerrig, 2005). In a study of audience design in language production, Horton and Gerrig (2005) showed that speakers more reliably tailored their referential expressions to the knowledge states of particular listeners when the experiences shared with the each listener were organized by category and thus easier to tell apart (e.g., one partner shared images of frogs, and another shared images of fish), as opposed to when the experiences shared with each listener were of the same kinds (both shared images of frogs and fish). This finding prompted us to provide hints such as the category and color cues in Experiment 2, to support children’s memory for what knowledge should be attributed to each interaction partner.

An open question is whether the interactive paradigm that allowed the children to freely interact with the speakers played a crucial role in our current findings. Adults sometimes show stronger sensitivity to contextual cues during live interactions than in non-interactive settings (Horton and Spieler, 2007; Brown-Schmidt, 2009; Yoon and Stine-Morrow, 2019). Children may also be more susceptible to cues made available through live interaction and if so, those cues could have been particularly advantageous in Experiment 2 when they needed to establish multiple representations of common ground. Interestingly, the studies that yield evidence for infants’ tracking of partner-specific common ground in offline tasks also rely on live interaction to create a memorable interaction history (e.g., Akhtar et al., 1996; Tomasello and Haberl, 2003).

Another open question is how much children benefited from the various hints we provided during the experimental interactions, and whether such supporting hints are necessary for young children to maintain and use partner-specific common ground in online comprehension. Young adults can establish and maintain multiple distinct representations even without such explicit hints to do so (see Yoon and Brown-Schmidt, 2018, 2019; Yoon and Stine-Morrow, 2019). When and how children develop the ability to routinely retain and use multiple representations of common ground remains an important open question regarding the development of partner-specific language processing. Interestingly, it may be that disfluency itself serves as a cue to the need to consult partner-specific common ground. Given the flexibility with which adult listeners interpret filled pauses, Barr and Seyfeddinipur (2010) speculated that *um*’s and *uh*’s might draw the listener’s attention to the knowledge and goals of the speaker. When a speaker is disfluent, the listener must infer the cause of the disfluency in order to interpret it appropriately as a cue for how the rest of the sentence might unfold. Disfluency could reflect the abilities of the speaker (as in the anomic or forgetful speakers described in the Introduction; Arnold et al., 2007; Orena and White, 2015), distraction by a concurrent task (Yoon and Fisher, 2020), or the conversational history shared with individual interaction partners, as in the present studies.

## Conclusion

Across two experiments, we investigated how children interpret disfluency when they interact with multiple partners who share different knowledge sets with them. We found that 4-year-old children were able to establish multiple representations of shared knowledge through live interaction, and then use this partner-specific information when processing a speaker’s disfluent referring expressions. Children treated disfluency as a predictor of reference to images that were novel and discourse-new from the perspective of the speaker rather than of the child.

## Data Availability Statement

The raw data supporting the conclusions of this article will be made available by the authors, without undue reservation.

## Ethics Statement

The studies involving human participants were reviewed and approved by the University of Illinois at Urbana-Champaign. Written informed consent to participate in this study was provided by the participants’ legal guardian/next of kin.

## Author Contributions

All authors contributed to planning and carrying out the experiments, writing the manuscript, and approved the final version of the manuscript for submission.

## Conflict of Interest

The authors declare that the research was conducted in the absence of any commercial or financial relationships that could be construed as a potential conflict of interest.

## Footnotes

1 If more than 25% of the time windows for a particular child were excluded, the participant was dropped from the analysis.
