# Supplementary material for: What’s New to You? Preschoolers’ Partner-Specific Online Processing of Disfluency
Source: Front Psychol. 2021 Jan 8;11:612601. doi: 10.3389/fpsyg.2020.612601 (PMC7820764; doi:10.3389/fpsyg.2020.612601)
Supplement: Supplementary file 1 [file Data_Sheet_1.pdf]

Supplementary Material

1 APPENDIX A: Model results

Statistical Analysis

In Experiments 1-2, the statistical analyses focus on the target advantage score during fluent and disfluent trials. Analyses of target advantage scores used Gaussian-link mixed effects models. In all analyses, models were fit using the lmer package in R, with the maximal random effects structure for subjects and items. In cases where the maximal model did not converge, a backwards-fitting procedure was used to identify the model with the largest random effects structure that would converge (see Barr, Levy, Scheepers, & Tily, 2013). Fixed effects were coded with mean-centered Helmert contrasts.

Table 1A. Experiment 1 target advantage scores in fluent trials: Mixed effect model with speaker type (same vs. different) and time window (pre-noun vs. noun) as fixed effects. The pre-noun window is treated as baseline. Values in bold indicate significant results.

	Estimate	SE	*t*-value	Pr(>|z|)			Variance	Std.Dev.
Fixed					*Random*			
(intercept)	0.99	0.36	2.76	0.07	*Subject*	(Intercept)	0.005	0.07
Speaker	-0.002	0.71	-0.003	1.00		Window	1.11	1.05
**Window**	-1.65	0.39	**-4.22**	**0.004**	*Trial*	(Intercept)	0.34	0.58
Speaker * Window	0.91	0.77	1.18	0.27		Speaker	1.34	1.15
						Window	0.14	0.37
						Speaker * Window	0.48	0.69
					Residual		4.10	2.03

Table 2A. Experiment 1 target advantage scores in disfluent trials: Mixed effect model with speaker type (same vs. different) and time window as fixed effects. Window was coded with two Helmert contrasts (Window1: pre-noun 1 and pre-noun 2 vs. noun; Window2: pre-noun 1 vs. pre-noun2). Values in bold indicate significant results.

	Estimate	SE	*t*-value	Pr(>|z|)			Variance	Std.Dev.
Fixed					*Random*			
(intercept)	-0.31	0.20	-1.54	0.18	*Subject*	(Intercept)	0.57	0.76
Speaker	-0.47	0.35	-1.35	0.18		Window 1	0.001	0.03
**Window 1**	-2.79	0.28	**-9.89**	**<.0001**		Window 2	0.66	0.81
Window 2	0.15	0.36	0.42	0.67	*Trial*	(Intercept)	0.04	0.21
Speaker * Window 1	0.99	0.56	1.76	0.08	*Residual*		4.82	2.20
Speaker * Window 2	-0.55	0.72	-0.76	0.45				

Table 3A. Experiment 2 target advantage scores in fluent trials: Mixed effect model with target familiarity (familiar vs. unfamiliar) and time window (pre-noun vs. noun) as fixed effects. The pre-noun window is treated as baseline. Values in bold indicate significant results.

<table>
<tr><th></th><th>Estimate</th><th>SE</th><th>t-value</th><th>Pr(>|z|)</th><th></th><th></th><th>Variance</th><th>Std.Dev.</th></tr>
<tr><td>Fixed</td><td></td><td></td><td></td><td></td><td>Random</td><td></td><td></td><td></td></tr>
<tr><td>(intercept)</td><td>2.12</td><td>0.16</td><td>12.87</td><td><.0001</td><td>Subject</td><td>(Intercept)</td><td>0.12</td><td>0.34</td></tr>
<tr><td>Target Familiarity (TF)</td><td>0.45</td><td>0.33</td><td>1.36</td><td>0.18</td><td></td><td>TF</td><td>0.98</td><td>0.99</td></tr>
<tr><td>Window</td><td>-2.18</td><td>0.30</td><td>-7.26</td><td><.0001</td><td></td><td>Window</td><td>1.08</td><td>1.04</td></tr>
<tr><td>TF * Window</td><td>-0.50</td><td>0.53</td><td>-0.95</td><td>0.35</td><td></td><td>TF * Window</td><td>3.15</td><td>1.77</td></tr>
<tr><td></td><td></td><td></td><td></td><td></td><td>Trial</td><td>(Intercept)</td><td>0.03</td><td>0.17</td></tr>
<tr><td></td><td></td><td></td><td></td><td></td><td></td><td>TF</td><td>0.04</td><td>0.20</td></tr>
<tr><td></td><td></td><td></td><td></td><td></td><td></td><td>Window</td><td>0.18</td><td>0.42</td></tr>
<tr><td></td><td></td><td></td><td></td><td></td><td></td><td>TF * Window</td><td>0.23</td><td>0.48</td></tr>
<tr><td></td><td></td><td></td><td></td><td></td><td>Residual</td><td></td><td>4.00</td><td>2.00</td></tr>
</table>

Table 4A. Experiment 2 target advantage scores in disfluent trials: Mixed effect model with target familiarity (familiar vs. unfamiliar) and time window as fixed effects. Window was coded with two Helmert contrasts (Window1: pre-noun 1 and pre-noun 2 vs. noun; Window2: pre-noun 1 vs. pre-noun2). Values in bold indicate significant results.

	Estimate	SE	*t*-value	Pr(>|z|)			Variance	Std.Dev.
Fixed					*Random*			
(intercept)	0.93	0.28	3.30	0.01	*Subject*	(Intercept)	<.0001	<.0001
**Target Familiarity (TF)**	**0.44**	**0.20**	**2.24**	**0.03**	*Trial*	(Intercept)	0.55	0.74
**Window 1**	-2.53	0.21	**-12.23**	**<.001**	*Residual*		5.04	2.24
Window 2	-0.19	0.24	-0.79	0.43				
TF * Window 1	-0.26	0.41	-0.63	0.53				
**TF * Window 2**	**1.02**	**0.49**	**2.10**	**0.04**				

2 APPENDIX B

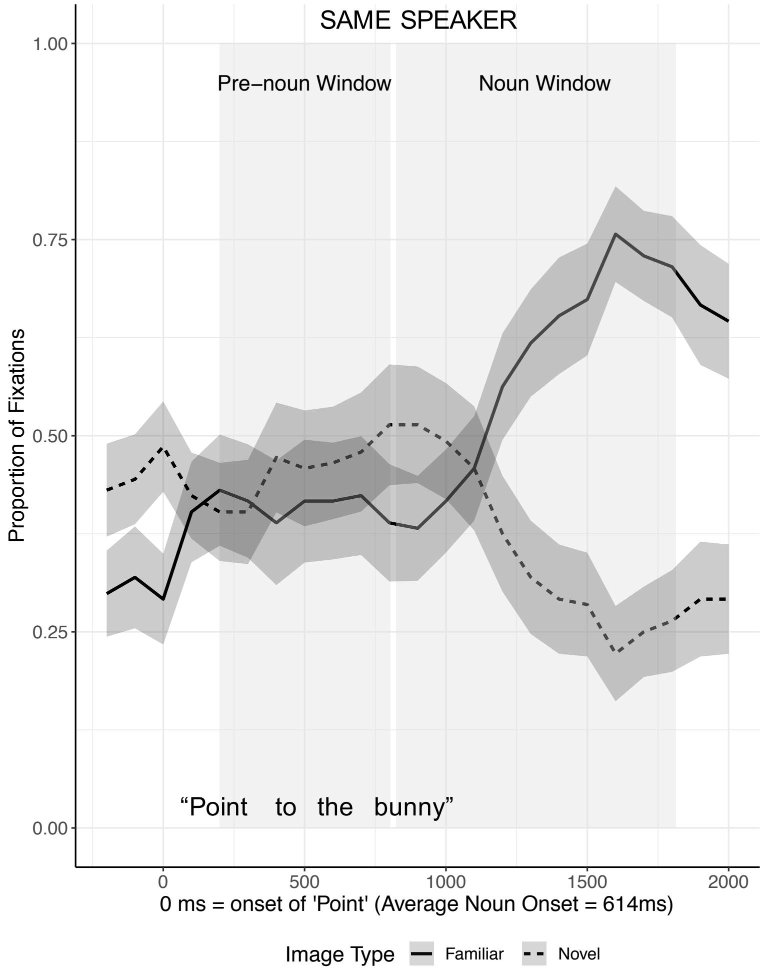

Figure 1B. Fluent trials in Experiment 1: Proportion of fixations following the onset of "Point", by Image Type (familiar vs. novel) in the Same Speaker condition.

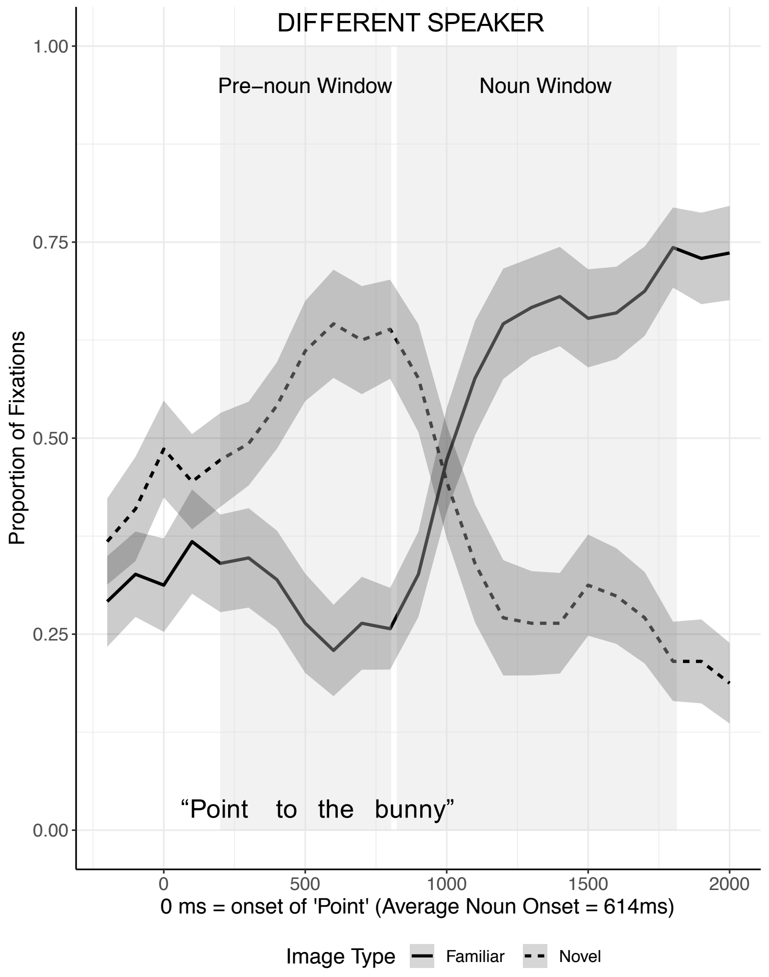

Figure 2B. Fluent trials in Experiment 1: Proportion of fixations following the onset of “Point”, by Image Type (familiar vs. novel) in the Different Speaker condition.

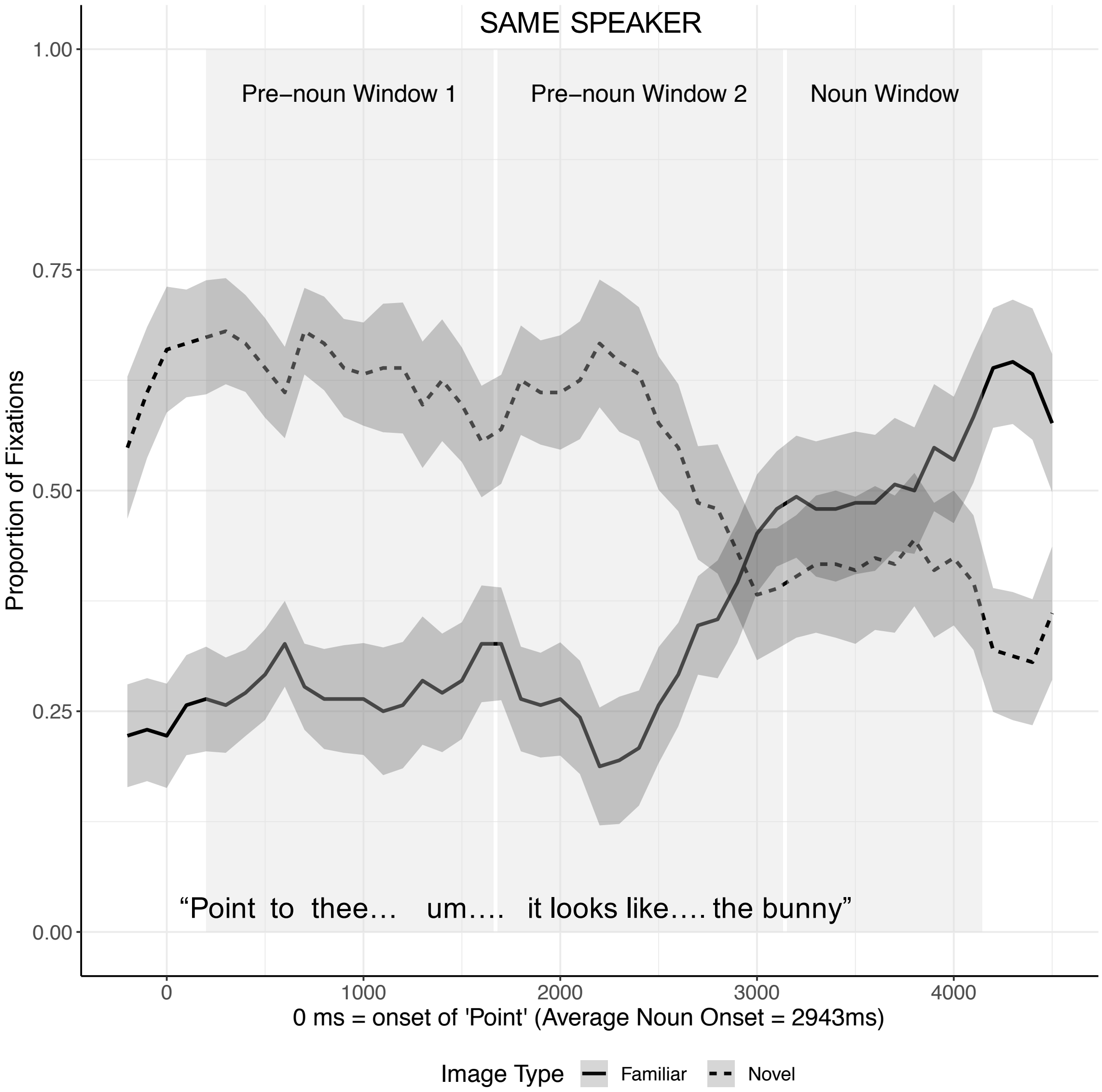

Figure 3B. Disfluent trials in Experiment 1: Proportion of fixations following the onset of “Point”, by Image Type (familiar vs. novel) in the Same Speaker condition.

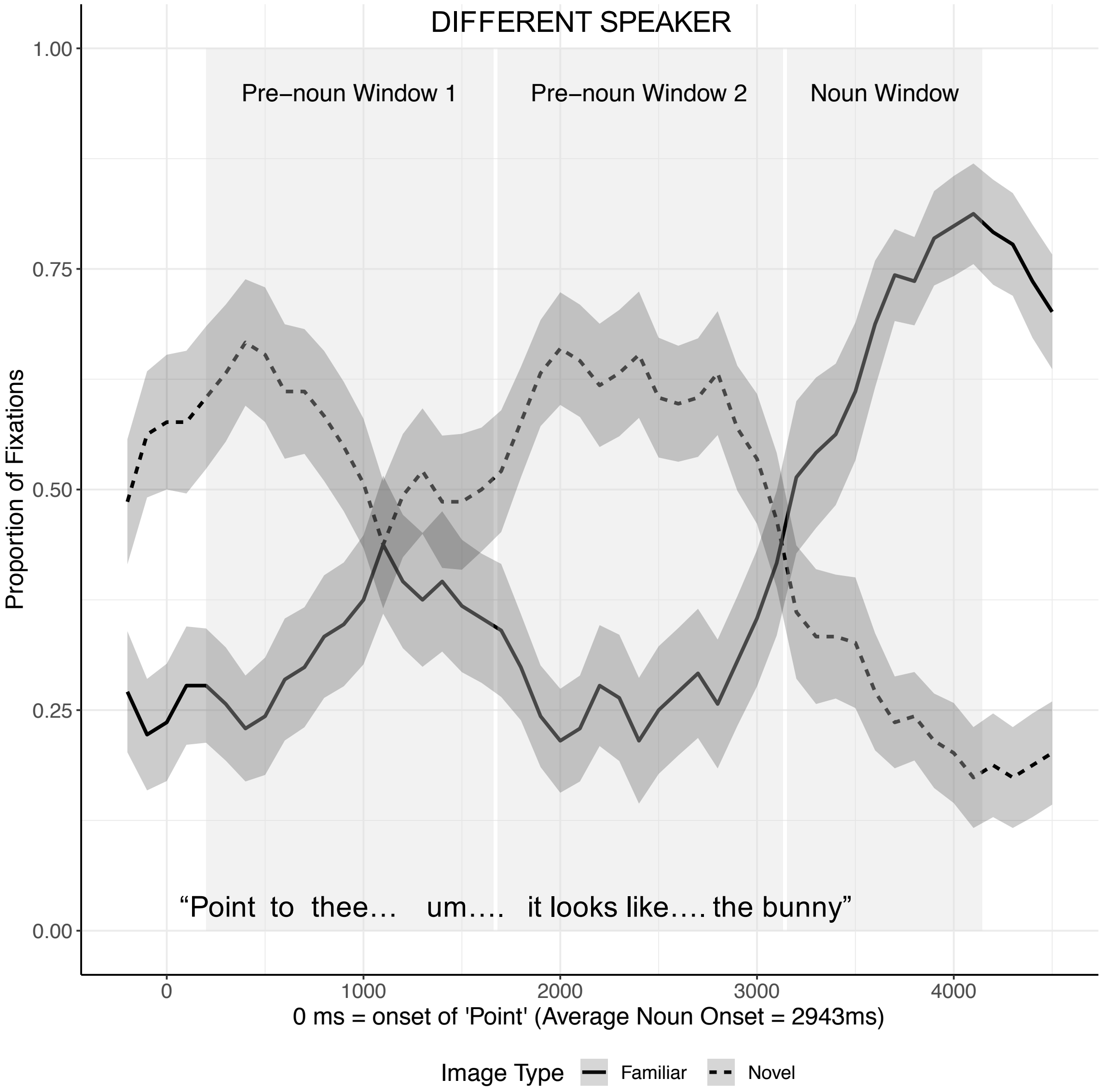

Figure 4B. Disfluent trials in Experiment 1: Proportion of fixations following the onset of "Point", by Image Type (familiar vs. novel) in the Different Speaker condition.

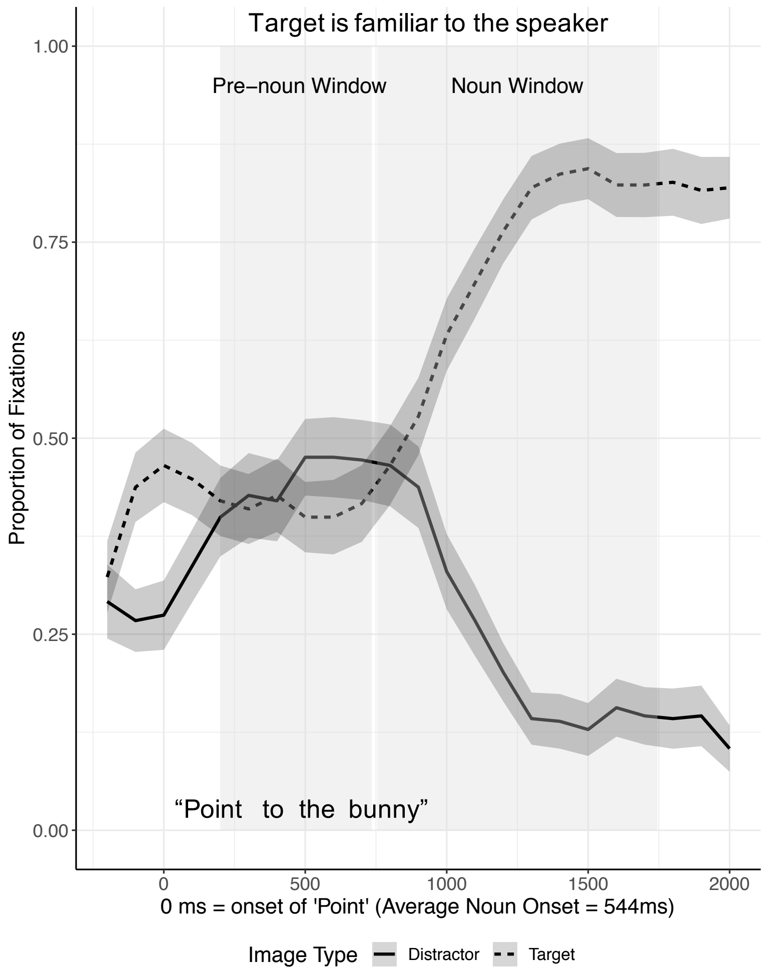

Figure 5B. Fluent trials in Experiment 2: Proportion of fixations following the onset of "Point", by Image Type (distractor vs. target) when the target is familiar to the speaker.

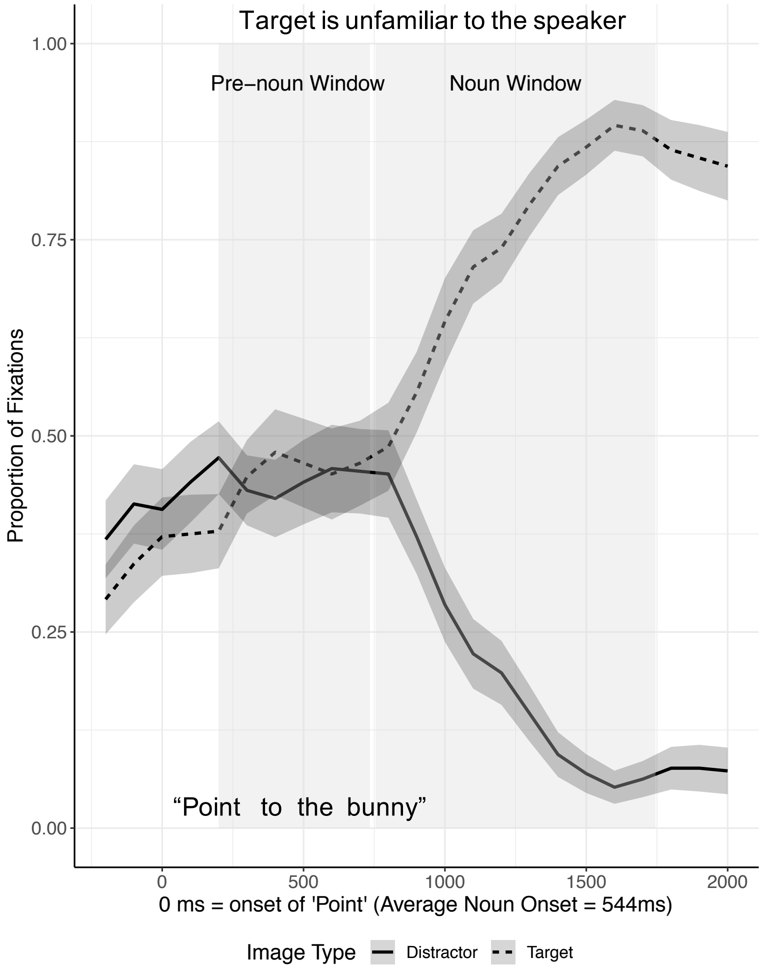

Figure 6B. Fluent trials in Experiment 2: Proportion of fixations following the onset of “Point”, by Image Type (distractor vs. target) when the target is unfamiliar to the speaker.

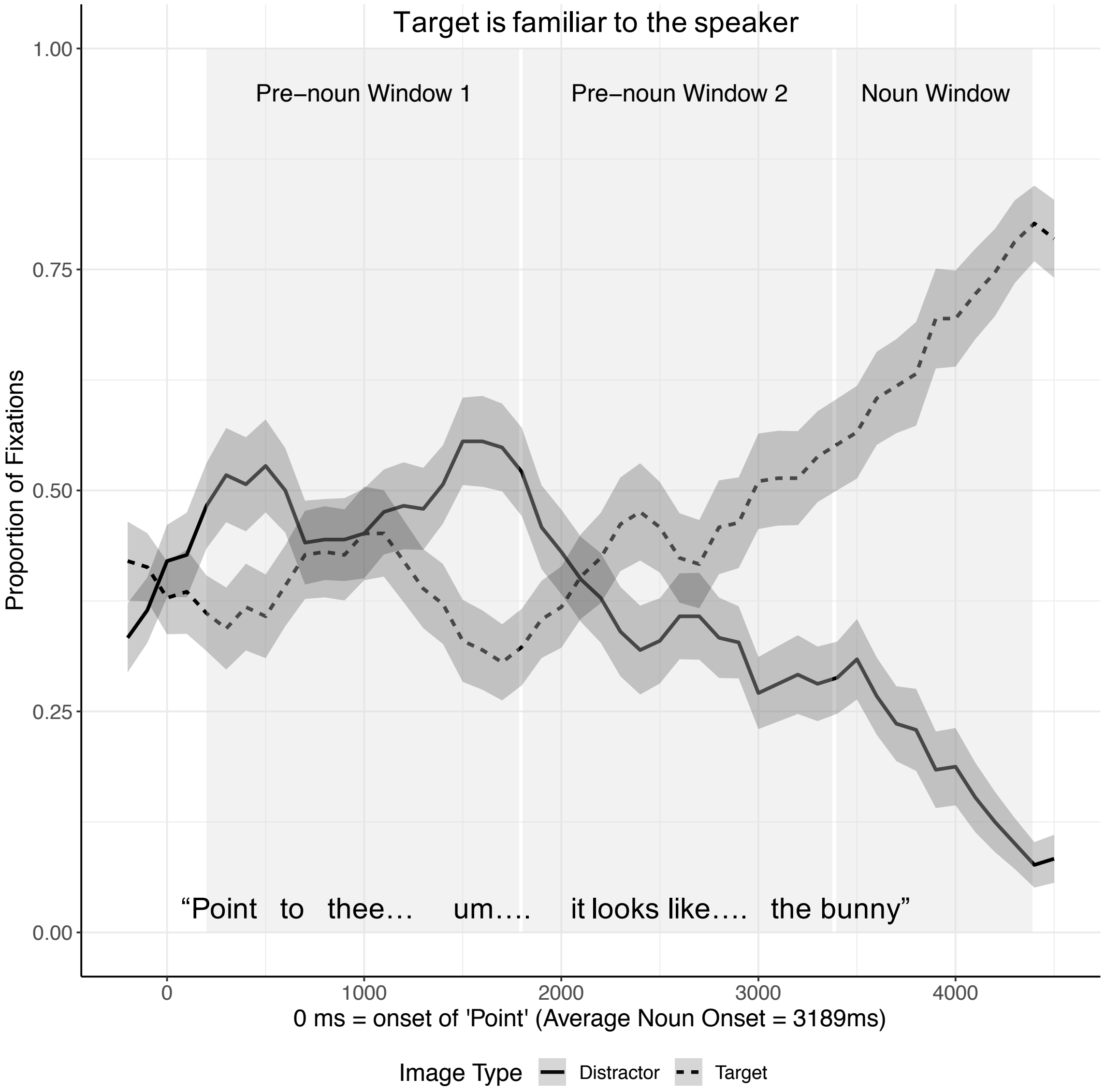

Figure 7B. Disfluent trials in Experiment 2: Proportion of fixations following the onset of “Point”, by Image Type (distractor vs. target) when the target is familiar to the speaker.

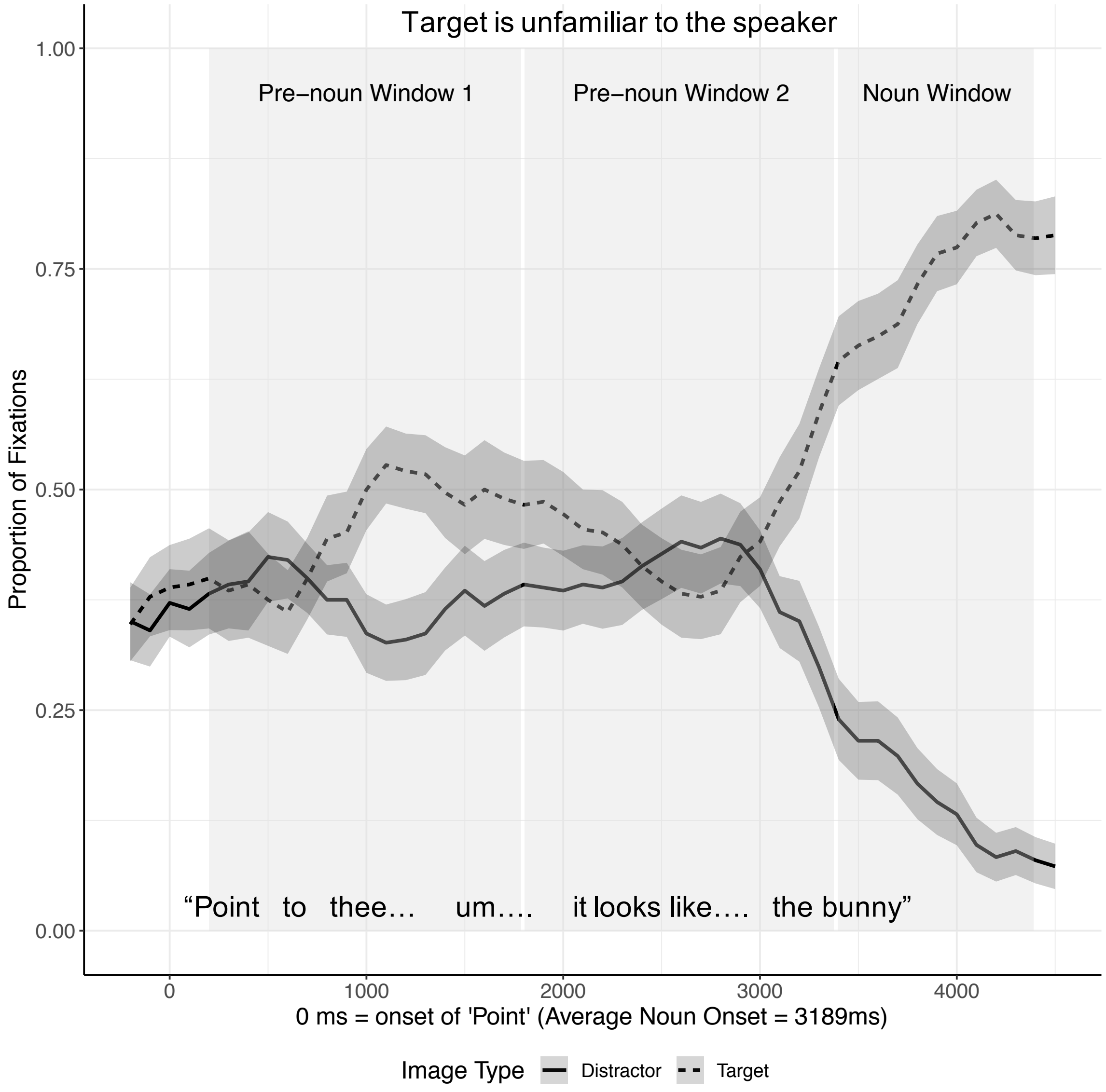

Figure 8B. Fluent trials in Experiment 2: Proportion of fixations following the onset of “Point”, by Image Type (distractor vs. target) when the target is unfamiliar to the speaker.
